# Supplementary figures and images for: Differentiating Epileptic and Psychogenic Non-Epileptic Seizures Using Machine Learning Analysis of EEG Plot Images
Source: Sensors (Basel). 2024 Apr 29;24(9):2823. doi: 10.3390/s24092823 (PMC11086151; doi:10.3390/s24092823)

**A**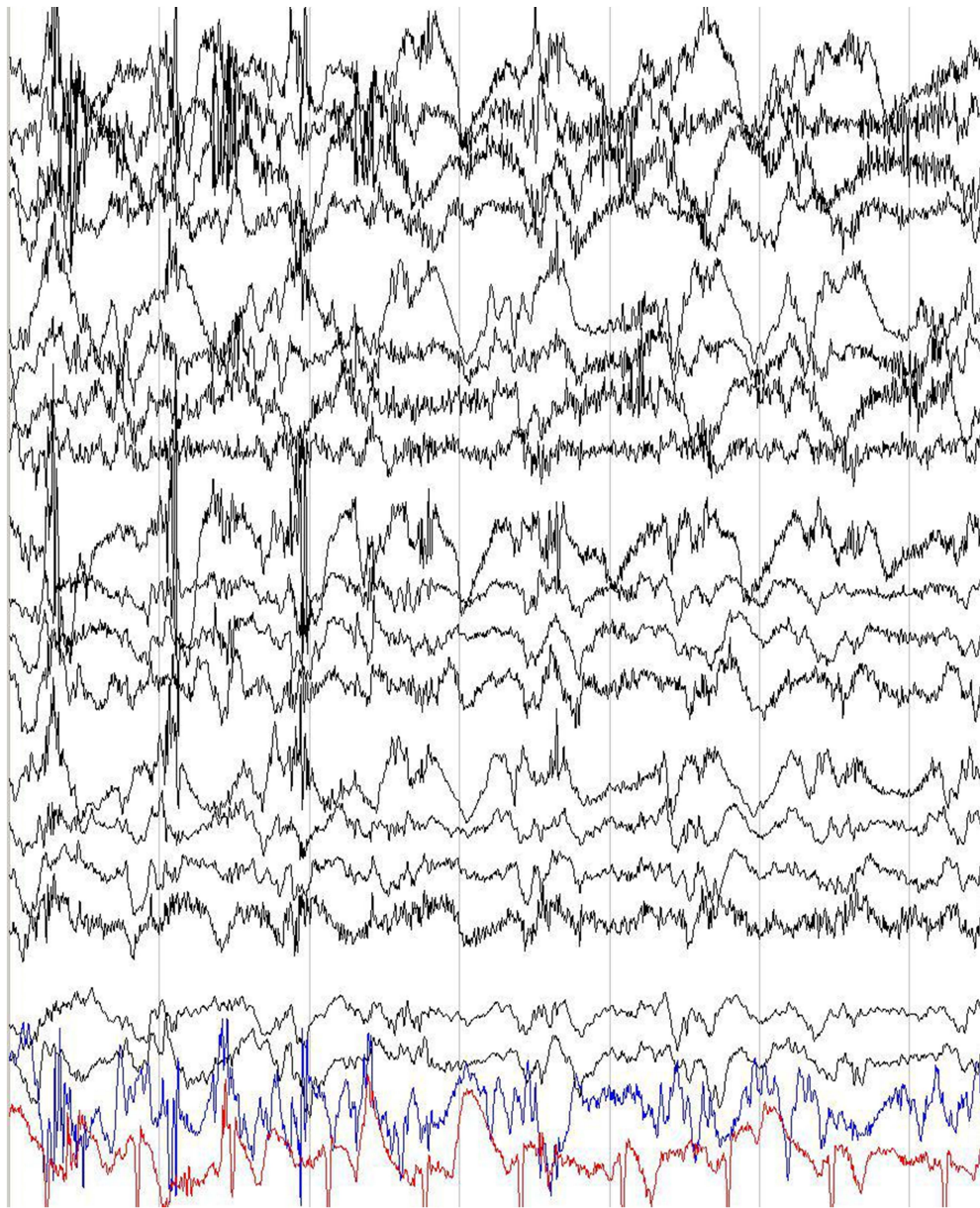**B**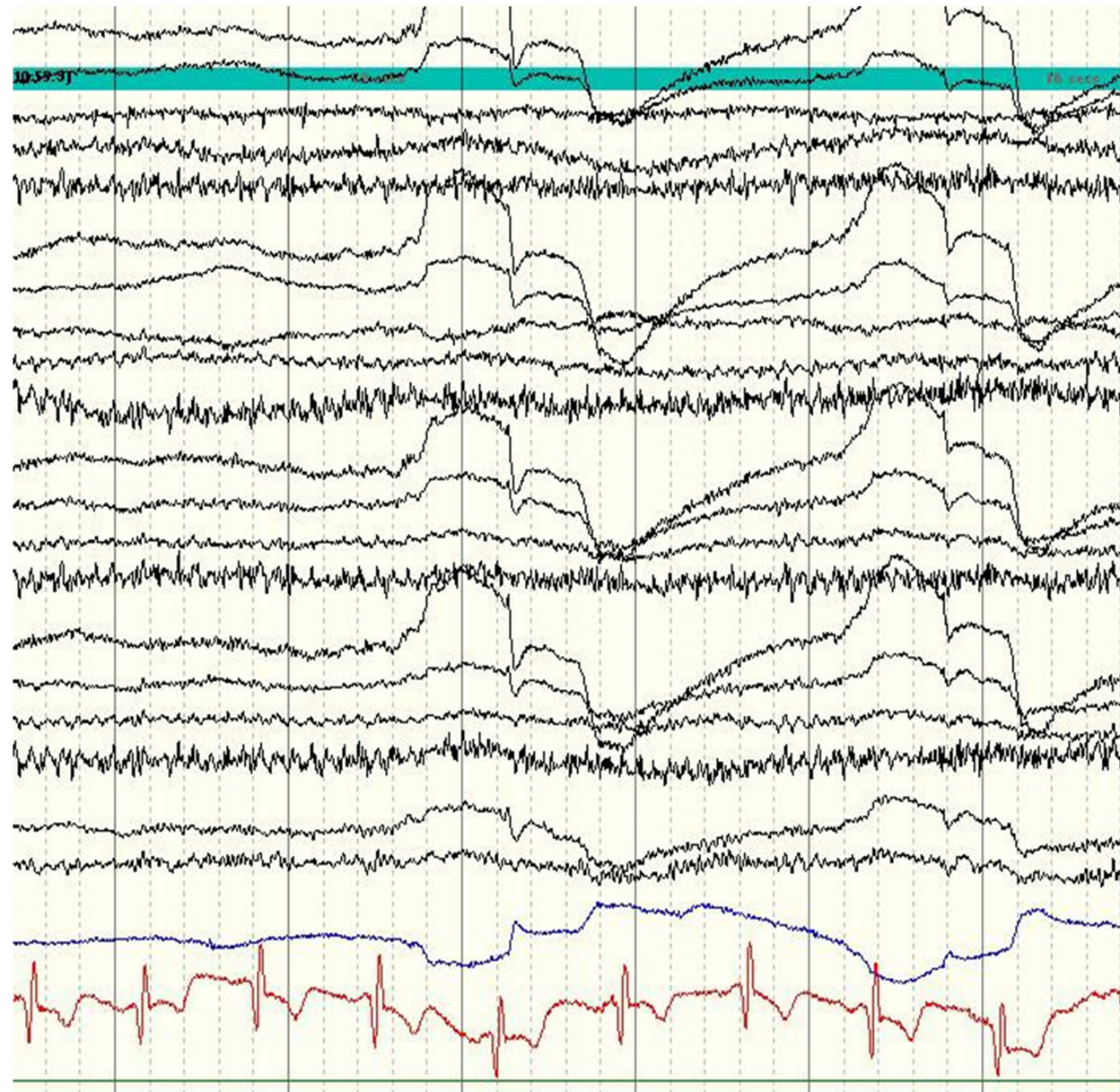

Supplement: Supplementary file 1 [file sensors-24-02823-s001.zip › sensors-2918681-supplementary.pdf]
